# NucMap: a database of genome-wide nucleosome positioning map across species

**DOI:** 10.1093/nar/gky980

**Published:** 2018-10-18

**Authors:** Yongbing Zhao, Jinyue Wang, Fang Liang, Yanxia Liu, Qi Wang, Hao Zhang, Meiye Jiang, Zhewen Zhang, Wenming Zhao, Yiming Bao, Zhang Zhang, Jiayan Wu, Yan W Asmann, Rujiao Li, Jingfa Xiao

**Affiliations:** 1 Department of Health Sciences Research, Mayo Clinic, Jacksonville, FL 32224, USA; 2 BIG Data Center, Beijing Institute of Genomics, Chinese Academy of Sciences, Beijing 100101, China; 3 CAS Key Laboratory of Genome Sciences and Information, Beijing Institute of Genomics, Chinese Academy of Sciences, Beijing 100101, China; 4 College of Life Sciences, University of Chinese Academy of Sciences, Beijing 100049, China; 5 Collaborative Innovation Center of Genetics and Development, Fudan University, Shanghai 200438, China

## Abstract

Dynamics of nucleosome positioning affects chromatin state, transcription and all other biological processes occurring on genomic DNA. While MNase-Seq has been used to depict nucleosome positioning map in eukaryote in the past years, nucleosome positioning data is increasing dramatically. To facilitate the usage of published data across studies, we developed a database named nucleosome positioning map (NucMap, http://bigd.big.ac.cn/nucmap). NucMap includes 798 experimental data from 477 samples across 15 species. With a series of functional modules, users can search profile of nucleosome positioning at the promoter region of each gene across all samples and make enrichment analysis on nucleosome positioning data in all genomic regions. Nucleosome browser was built to visualize the profiles of nucleosome positioning. Users can also visualize multiple sources of omics data with the nucleosome browser and make side-by-side comparisons. All processed data in the database are freely available. NucMap is the first comprehensive nucleosome positioning platform and it will serve as an important resource to facilitate the understanding of chromatin regulation.

## INTRODUCTION

Eukaryotic genomic DNA is tightly packaged into compacted nucleosome arrays, which are the fundamental units of chromatin structure (1). The term ‘nucleosome positioning’ is widely used to indicate where nucleosomes occupy on genomic DNA sequence (2–4). In the nucleus, nucleosomes dynamically transform between depletion and de novo occupation on genomic DNA, affecting all biological processes occurring on genomic DNA (5–7). It has been further reported that nucleosome positioning affects transcription initiation and elongation (8). Transcriptional machinery must access to chromatin to trigger sequential gene transcription (7), while nucleosome organization can influence gene activity by controlling the accessibility of transcriptional factor binding sites (9). Some studies suggested that nucleosome positioning influences the evolution of DNA sequence (10–12) since DNA repair machinery has different preferential access between linker DNA and nucleosomal DNA (13).

Until now, many different methods have been developed to mapping nucleosomes, such as predicting nucleosome positioning based on DNA sequence features (14,15), histone ChIP-Seq (16), or chromatin accessibility profiles (17). However, all these methods have limitation on either resolution or genome-wide coverage. MNase-Seq is another prevalent experimental approach in nucleosome mapping. In this approach, chromatin is digested with micrococcal nuclease and then followed by deep sequencing (18,19). Based on MNase-Seq, many computational tools have been developed to facilitate the application of this technology (20). Several programs have been published to identify nucleosome peaks, such as DANPOS (21) and iNPS (22). To better understand the role of nucleosome, it is very important to compare nucleosome profiles across different conditions or cell types. Multiple tools were developed to identify differential nucleosome regions, such as DANPOS (21), DiNuP (23), and Dimnp (24). In the past years, a large number of studies has employed MNase-Seq to depict nucleosome positioning map in eukaryote ranging from yeast to human (5,18,25,26). Consequently, MNase-Seq data is rapidly growing across a wide variety of organisms. It is imperative to build a platform to collect and integrate all published data and make datasets from different studies reusable and comparable, which will largely help biologist to further understand the biology behind nucleosome positioning. However, no such a database or platform was reported. To fill this gap, here we present NucMap, a database of genome-wide nucleosome positioning map across species. Based on a large collection of raw sequence data from published studies, NucMap is dedicated to integrating, analyzing, and visualizing nucleosome positioning data across species.

## DATABASE IMPLEMENTATION

All raw MNase-Seq data were downloaded from GEO and ENCODE, processed by in-house pipeline, and then imported into the NucMap database. The main framework of NucMap was developed based on PHP and MySQL, which are a popular and open source script language and a relational database management system for web development, respectively. JQuery and Bootstrap were used to design the front-end web interface. AJAX (Asynchronous JavaScript And XML), a set of Web development techniques, was used to create asynchronous bioinformatics application running in the back-end. Back-end bioinformatics applications were implemented with Python and Bash. JBrowser (27) was integrated to visualize nucleosome positioning data.

## DATABASE CONTENT AND USAGE

### Overview of NucMap

Currently, we have collected and processed 798 experimental datasets from 477 samples across 15 species. All functionalities in NucMap are organized into four modules: search, browse, analysis and download.

### Searching NucMap

**Figure 1.**
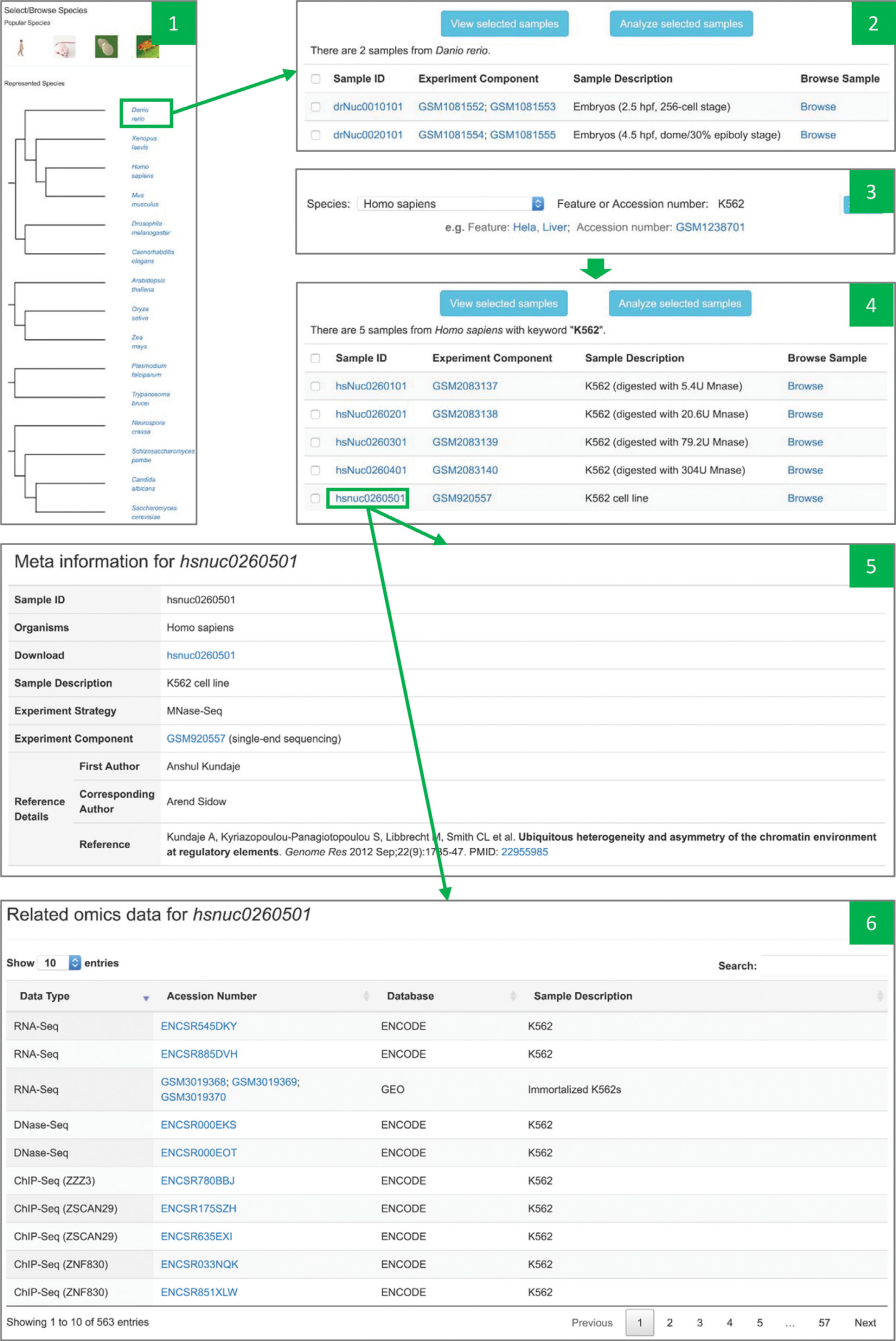
Screenshots of sample-based search module. Panel 1: interface of shortcut links for all species and popular species. Panel 2: searching result by clicking the shortcut link for ‘*Danio rerio*’. Panel 3: the interface for keyword-based search. Panel 4: result by searching with ‘K562’ in ‘*Homo sapiens*’. Panel 5: the snapshot of sample meta information for hsnuc0260501. Panel 6: the snapshot of related omics data for hsnuc0260501 (only partially datasets were shown).

We have developed two types of search modules in NucMap, which are sample-based and gene-based. Sample-based search mainly focuses on helping users to find the samples they are interested in (Figure 1). Shortcut links can be used to obtain all samples for specific species. Users can also search sample of interest with accession number in GEO and ENCODE or sample feature. With hyperlinked sample ID, users can access more specific information for each sample, including original data source, original publication, all downloadable data for this sample and other related omics samples. On the page of search result, the buttons ‘View selected samples’ and ‘Analyzed selected samples’ connect searching results to other modules in NucMap. With these two buttons, users can visualize nucleosome positioning data from selected samples in the nucleosome browser or perform analysis on selected samples with the online analysis module, which will be introduced in the later sections.

**Figure 2.**
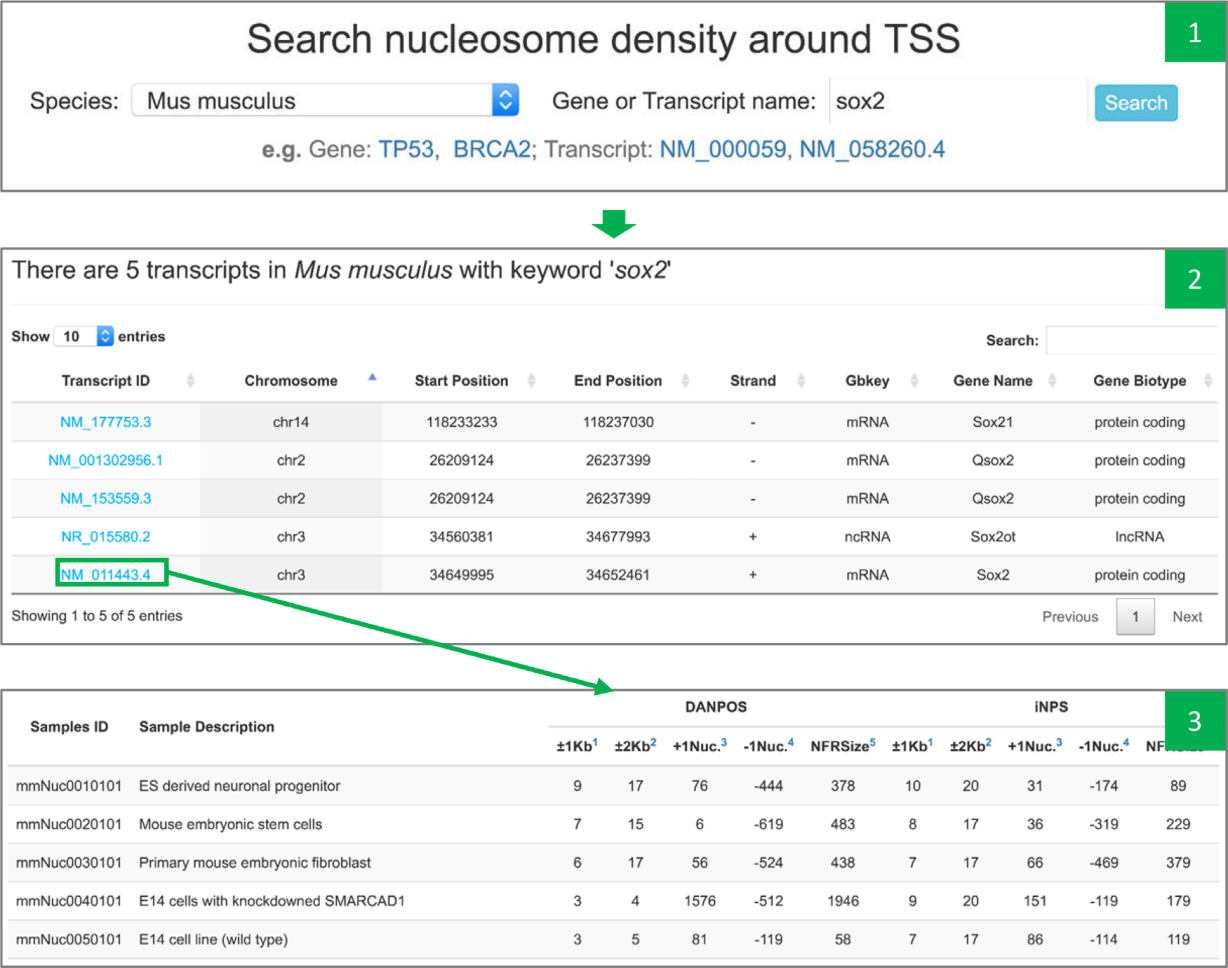
Screenshots of gene-based search module. Panel 1: interface for gene/transcript searching. Panel 2: all available transcripts in *Mus musculus* with the keyword ‘sox2’. Panel 3: peak number and nucleosome positioning profile at the transcription start site of NM_011443.4.

Promoter-associated nucleosome free region (NFR) is related to promoter-proximal pausing to enable precise gene regulation (28,29). Gene-based searching helps users to checkup nucleosome positioning at the promoter region of an individual gene. Both gene name and transcript name are supported in searching. The number of nucleosome peaks at transcription start site were provided in different genomic ranges. The positions of +1 and −1 nucleosome are provided to check up the size of NFR at promoter. Moreover, nucleosome density information across all samples in the same species will be shown in the same table, so that users can make side-by-side comparison (Figure 2; Table 1).

**Table 1.** The number of experiments and samples in each species

Species	#Experiment	#Samples
*Arabidopsis thaliana*	19	12
*Caenorhabditis elegans*	21	11
*Candida albicans*	4	2
*Danio rerio*	4	2
*Drosophila melanogaster*	124	70
*Homo sapiens*	71	50
*Mus musculus*	215	106
*Neurospora crassa*	10	6
*Oryza sativa*	3	1
*Plasmodium falciparum*	9	9
*Saccharomyces cerevisiae*	284	186
*Schizosaccharomyces pombe*	18	14
*Trypanosoma brucei*	8	4
*Xenopus laevis*	6	2
*Zea mays*	2	2

### Nucleosome browser

**Figure 3.**
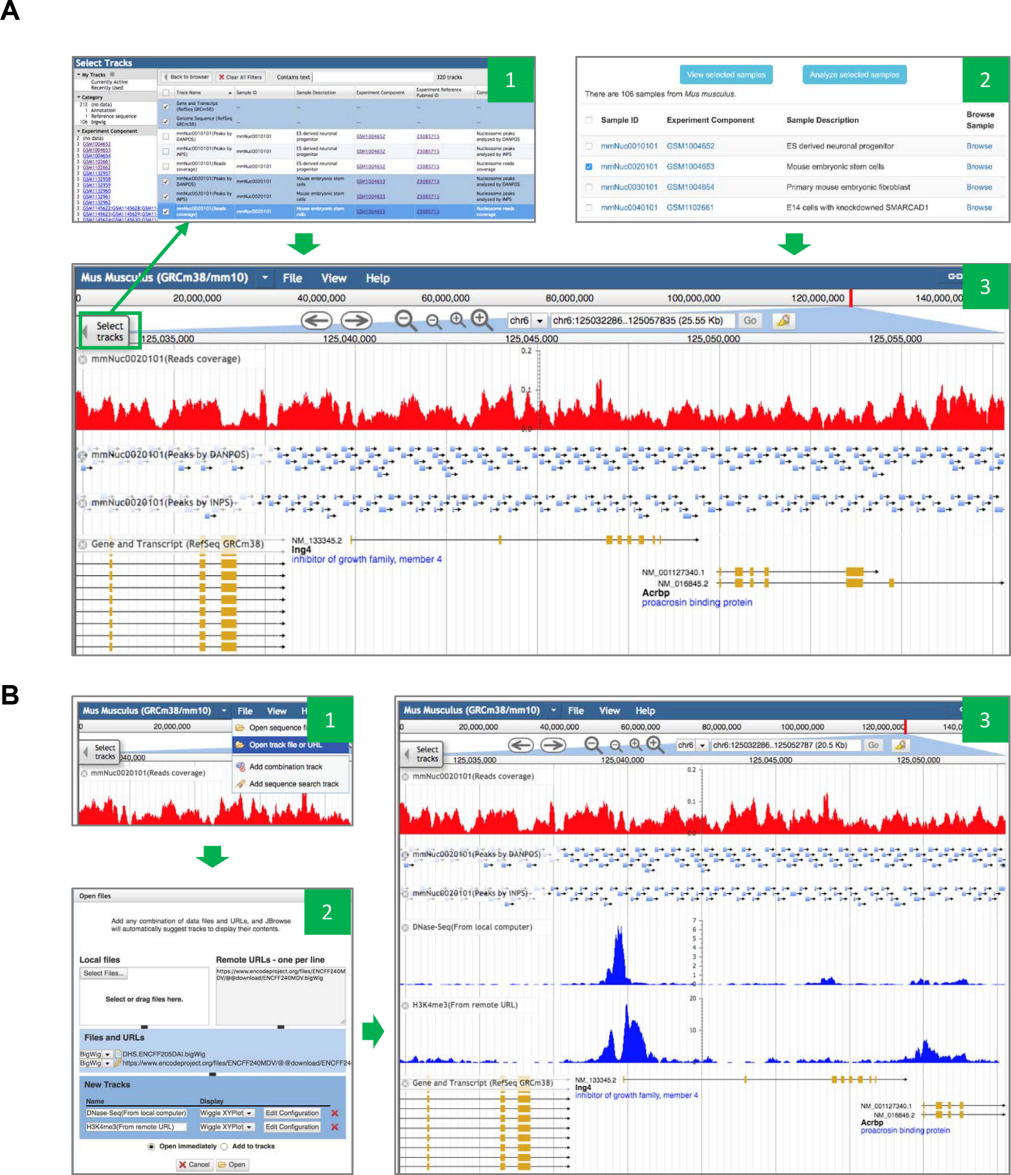
Screenshots of nucleosome browser. (**A**) Browsing tracks deposited the NucMap database. There are two routes to choose and browse sample tracks of interest, which are shown as Panel 1 and 2 respectively. Panel 1: choose tracks of interest with the track selection table in the nucleosome browser. Panel 2: click ‘Browse’ link associated to each sample or click ‘View selected sample’ button after selecting sample of interest in sample-based search module. Panel 3: visualizing tracks from the sample ‘mmNuc0020101’, in which green box is the button to show track selection table. (**B**) Browsing tracks from local computer or remote URL. Panel 1: menu for opening a track file or remote URL. Panel 2: interface for loading and configuring tracks from local computer and remote URL. Panel 3: visualizing tracks from local computer and remote URL (blue) together with tracks from the sample ‘mmNuc0020101’.

To facilitate browsing nucleosome profile at single-base resolution, NucMap has deployed a nucleosome browser based on the open source program JBrowser. In the nucleosome browser, each species has an independent browser instance and track selector. With track selector, users can load or unload the tracks for all processed genomic data, including raw reads density and nucleosome peaks analyzed by different algorithms (Figure 3A). With interactive buttons and interfaces, users can choose tracks of interest, and zoom in/out and highlight any genomic region on the whole genome. This feature will help users to check every detail regarding nucleosome occupancy on each individual gene or genomic region. Meanwhile, users can also directly load track files from their local computer or a third-part database to the nucleosome browser without uploading data to our server (Figure 3B). Therefore, nucleosome browser can help users quickly make side-by-side comparison across multiple relevant genomic track files. For example, biologist can load DNA methylation data or histone ChIP-Seq data into the same browser session and obtain a comprehensive overview of chromatin state around a gene of interest.

### Analysis

**Figure 4.**
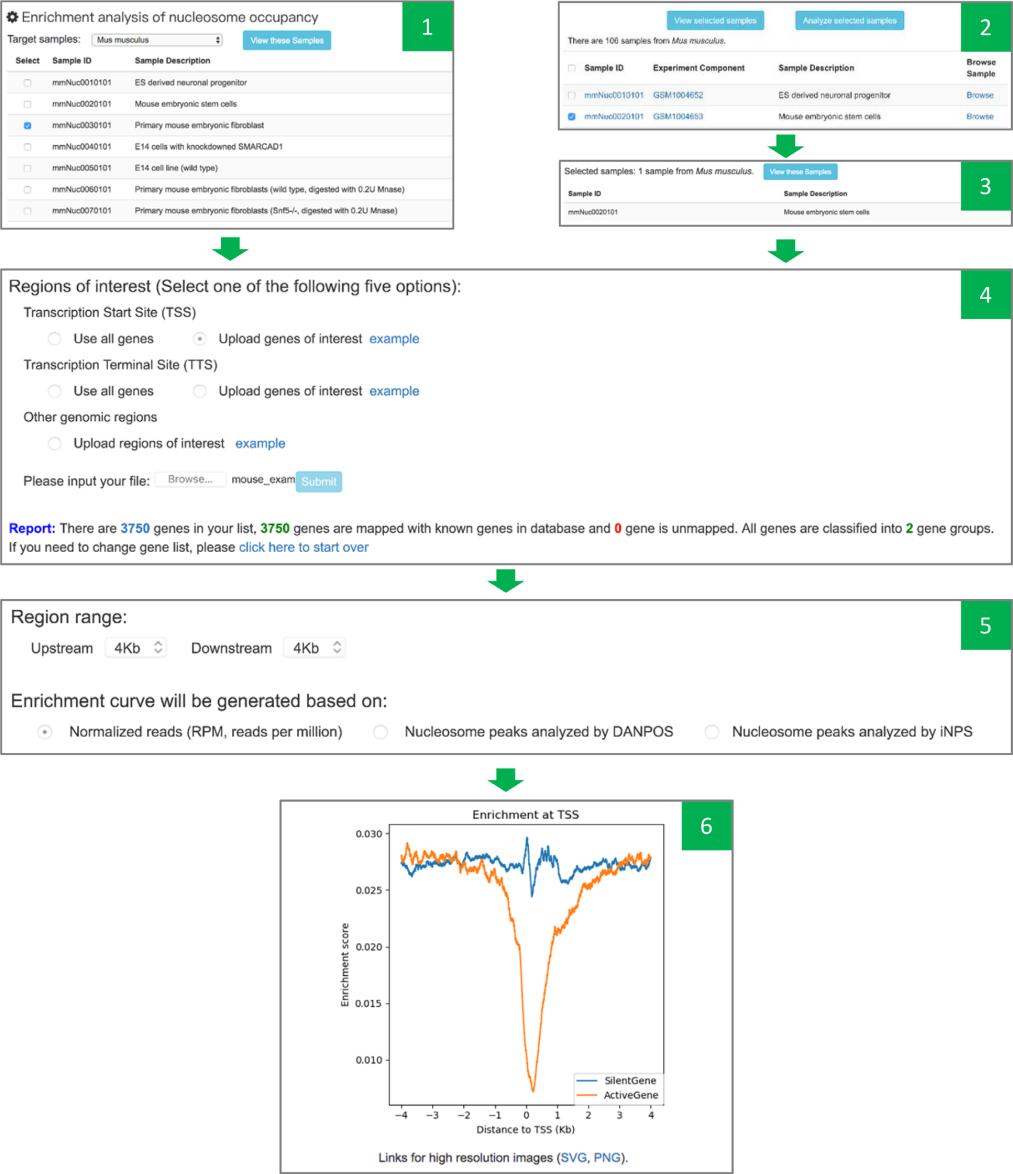
Screenshots of online enrichment analysis. There are two routes to choose samples of interest: choosing samples on the analysis module page (Panel 1) and choosing samples on the sample searching page (shown as Panel 2 and Panel 3). Panel 4 is showing the interface for selecting regions of interest, uploading file for genes or regions of interest, and validity report of uploaded genes or genomic regions. Panel 5 is the interface for analysis parameter selection. Panel 6 is the figure automatically generated by the online analysis module and the links for high resolution figures.

Genome-wide enrichment analysis is a popular method to understand global features in omics data. To help users make a global analysis on nucleosome positioning patterns, we have developed online analysis module. This module can characterize nucleosome occupancy profile at all genomic regions (Figure 4). Users can also classify the regions of interest into multiple groups according to the purpose of their studies, so that they can compare the difference of enrichment curves among groups. Both normalized raw reads and nucleosome peaks are supported in the enrichment analysis. Finally, publication-quality figures will be presented. All operations are based on web interface, and there is no requirement for prior knowledge regarding bioinformatics tools and programming.

### Download

All processed nucleosome positioning data are freely available. The data for each sample include three levels; (i) processed reads: bigwig track based on aligned reads and aligned reads after enhancing signal; (ii) nucleosome peaks: nucleosome peaks identified by iNPS and DANPOS; (iii) annotated peaks and reads: nucleosome peaks annotated to nearest TSS, the matrix of peak count around each TSS and the matrix of aligned reads count around each TSS, which were normalized to RPM (Reads Per Million). For each species, all these data were organized in two ways on the download page, by sample and by data type. Users can also visualize our data with our online links in their local browser or other online genome browsers.

## FUTURE DIRECTIONS

MNase-Seq is an important approach to study the role of nucleosome in transcriptional regulation. With an increasing usage of MNase-Seq in eukaryotes, nucleosome positioning data is rapidly growing. NucMap is the first open resource and platform for nucleosome positioning data from MNase-Seq across species. All available MNase-Seq data in GEO and ENCODE up to date are included in NucMap. As one of important database resources in BIG Data Center (30), NucMap will be continuously collecting and integrating published data.

Nowadays, biologists usually integrate and analyze multiple-scales omics data to study transcriptional regulation. Nucleosome positioning is one type of chromatin state information. To deeply understand chromatin biology, we will make NucMap compatible to other public epigenomics databases, such as MethBank (31), Cistrome (32) and ENCODE (33). Thus, datasets in other repositories, such as DNA methylation data, histone and transcription factor ChIP-Seq, can be directly loaded and compared with nucleosome positioning data in NucMap. Based on comprehensive analysis on cross-omics data, biologists will therefore learn more about chromatin regulation.
